# Contactless Fatigue Level Diagnosis System Through Multimodal Sensor Data

**DOI:** 10.3390/bioengineering12020116

**Published:** 2025-01-26

**Authors:** Younggun Lee, Yongkyun Lee, Sungho Kim, Sitae Kim, Seunghoon Yoo

**Affiliations:** 1 Department of Electronics and Communication Engineering, Republic of Korea Air Force Academy, 635 Danjae-ro, Sangdang-gu, Cheongju 28187, Republic of Korea; 2 Department of Mathematics, Republic of Korea Air Force Academy, 635 Danjae-ro, Sangdang-gu, Cheongju 28187, Republic of Korea; mathyouth@gmail.com; 3 Department of Systems Engineering, Republic of Korea Air Force Academy, 635 Danjae-ro, Sangdang-gu, Cheongju 28187, Republic of Korea; dilemma37@naver.com; 4 Department of Mechanical Engineering, Republic of Korea Air Force Academy, 635 Danjae-ro, Sangdang-gu, Cheongju 28187, Republic of Korea; sitaikim@gmail.com; 5 Department of Computer Science, Republic of Korea Air Force Academy, 635 Danjae-ro, Sangdang-gu, Cheongju 28187, Republic of Korea

**Keywords:** fatigue level, pre-mission, contactless, AI-classifier, multimodal, task performance, personalized, non-invasive

## Abstract

Fatigue management is critical for high-risk professions such as pilots, firefighters, and healthcare workers, where physical and mental exhaustion can lead to catastrophic accidents and loss of life. Traditional fatigue assessment methods, including surveys and physiological measurements, are limited in real-time monitoring and user convenience. To address these issues, this study introduces a novel contactless fatigue level diagnosis system leveraging multimodal sensor data, including video, thermal imaging, and audio. The system integrates non-contact biometric data collection with an AI-driven classification model capable of diagnosing fatigue levels on a 1 to 5 scale with an average accuracy of 89%. Key features include real-time feedback, adaptive retraining for personalized accuracy improvement, and compatibility with high-stress environments. Experimental results demonstrate that retraining with user feedback enhances classification accuracy by 11 percentage points. The system’s hardware is validated for robustness under diverse operational conditions, including temperature and electromagnetic compliance. This innovation provides a practical solution for improving operational safety and performance in critical sectors by enabling precise, non-invasive, and efficient fatigue monitoring.

## 1. Introduction

Modern society operates in increasingly complex and demanding environments, highlighting fatigue management and safety issues in specific professions as critical societal challenges [1,2,3,4]. Occupations such as pilots, firefighters, and nurses face intense physical and mental demands, where fatigue not only impacts individual health but also poses severe risks of large-scale accidents and loss of life [5,6,7]. Fatigue in these roles is a major factor that degrades performance, increases accident rates, and endangers both lives and property [8,9,10].

Traditional fatigue assessment methods, relying on surveys [11,12,13,14] or physiological signal measurements, are often limited by their inability to provide real-time monitoring or by causing discomfort to users [15]. In this context, non-invasive multi-sensor technologies have emerged as promising alternatives, capable of minimizing physical discomfort while enabling precise, real-time data collection and analysis for fatigue evaluation [15,16,17,18].

This paper proposes a novel fatigue assessment system using non-contact multi-sensor data targeting high-risk occupations where fatigue levels directly impact safety and large-scale accident prevention. The main contributions of this paper are fourfold:

1 The system provides immediate fatigue diagnosis for healthy individuals.
2 It leverages non-contact sensors to collect short-term data and utilizes an AI-based fatigue classifier for highly accurate diagnostics.
3 The system evaluates pre-mission fatigue levels, improving operational safety and reducing the risk of fatalities in high-risk professions.
4 With repeated fatigue diagnostics and cumulative data, the system enables retraining for even more precise diagnostic results over time.

The rest of this paper is organized as follows: Section 2 reviews related research, while Section 3 elaborates on the design and architecture of the proposed system. Section 4 describes the hardware, software, and fatigue classification model. Section 5 presents the experimental results and performance evaluation. Finally, the paper concludes with Section 6.

## 2. Related Work

Fatigue is a normal and common experience resulting from mental focus, lack of sleep or rest, or physical exertion [19,20,21,22,23]. It may also occur when individuals lack motivation to achieve their tasks [1,24,25,26,27]. Although fatigue is a concept that lacks clear definition, it can be categorized into two types: subjective fatigue, often referred to as central or mental fatigue, and task-induced fatigue, which is assessed based on the individual’s ability to maintain physical alertness suitable for task performance [8,28,29,30,31,32].

To explore mild fatigue levels that affect task performance rather than pathological fatigue, existing measurement methods include self-reported surveys [11,12,13,14,33,34,35], physiological tests [36,37,38], cognitive-behavioral evaluations [39,40,41], and biochemical methods [2,15,42,43,44]. These methods, however, are not absolute in classifying fatigue levels. Self-reported methods rely on subjective evaluations, which are inherently limited in objectivity, while physiological measures are subject to significant individual variability, making it challenging to establish universal thresholds for fatigue classification [5,45,46,47,48].

Cognitive-behavioral assessments can classify fatigue levels by evaluating alertness, yet they primarily measure indirect fatigue levels and are susceptible to learning effects, making results less reliable [2,39,40,41]. Additionally, such assessments may be influenced by temporary focus or motivation, potentially distorting outcomes.

Biochemical methods are advantageous in that they cannot be intentionally altered by the test subject and typically exhibit lower variability [2,15,42,43,44]. However, no single biochemical marker fully reflects fatigue, and combinations of markers are often required depending on individual circumstances [8]. These methods are also costly and time-consuming, which limits their practical application. Therefore, single-method evaluations face inherent limitations in extracting ground truth fatigue levels, and current fatigue assessments often involve a combination of methods for fatigue and risk management [1,49,50,51,52,53].

To date, the most reliable tools for assessing fatigue include multidimensional fatigue scales based on self-reported surveys, contact-based sensor measurements (e.g., electromyography or respiratory sensors), and invasive methods such as lactate or hormone analysis in blood samples [12,15,54,55,56,57,58,59,60,61]. However, these methods are cumbersome, time-intensive, and impractical for on-site application, prompting a need for non-contact and time-efficient fatigue assessment methods.

This paper proposes a novel approach: collecting optical and thermal imaging along with voice signals using non-contact devices and employing an AI-based classifier to measure fatigue and provide real-time feedback to the user. This approach targets high-risk occupations such as long-distance truck and bus drivers, confined-space workers, and firefighters, where accumulated fatigue could lead to catastrophic accidents. Accurate fatigue assessment aims to facilitate task scheduling adjustments and ensure adequate rest, thereby preventing accidents.

## 3. System Design and Architecture

This section introduces the overall concept of operations for the system and describes the required system specifications, divided into hardware and software components. Additionally, it provides an explanation of the dataset used to implement the fatigue classifier model.

### 3.1. Concept of Operations

**Figure 1:**
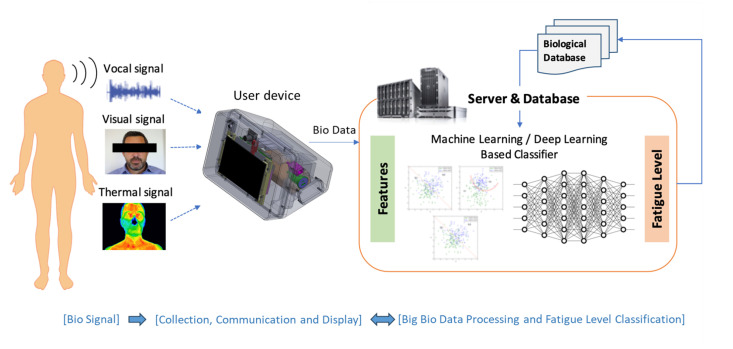
Overview of the fatigue diagnosis system.

The fatigue level diagnosis system comprises a biometric data collector, an AI-based fatigue classification model, and an integrated platform for system management and monitoring, as shown in Figure 1.

Our proposed system in this paper is designed primarily to assess “pre-mission fatigue”. Its goal is to determine whether a user is fit to perform a mission by measuring fatigue levels before starting the task. The biometric data collector is a device that gathers data from users (e.g., pilots or workers) in real time and in a non-contact manner, providing instant fatigue level results. For uniform data collection, the collector is placed on a desk within 1 m of the user, ensuring the user’s face is visible on the device’s screen. To enable individual fatigue tracking and management, users log in with their ID and password. Data collection occurs in two stages: in the first stage, users read a long sentence, and in the second stage, they read a short sentence. During each stage, the system collects video, audio, and thermal imaging data while the user reads the provided text. The collected data are then transmitted to a remote integrated platform.

The integrated platform uses the transmitted biometric data (video, audio, and thermal imaging) and the fatigue classification model to immediately diagnose fatigue levels on a scale of 1 to 5. The diagnosis results are sent back to the biometric data collector, which displays them on the screen. Users can then view their fatigue levels and provide feedback by selecting their perceived fatigue level on a scale of 1 to 5. The collector transmits this feedback to the integrated platform for personalized learning, further improving the AI model’s accuracy for each individual.

### 3.2. System Requirements

#### 3.2.1. Hardware

The system hardware consists of a data collector equipped with multiple sensors and a server responsible for diagnosing fatigue levels based on the transmitted data. The collector must be equipped with sensors capable of collecting non-contact biometric signals, including video, audio, and thermal imaging data. It should enable fatigue measurement at a distance of 1 m between the user and the collector. This is because typical usage scenarios involve portable devices with cameras and screens, where the user is typically positioned about 1 m away from the device. For seamless communication with the server, the device must support internet connectivity via both Wi-Fi and Ethernet. Additionally, the collector should be operable for more than 2 h using battery power, alongside standard AC power supply.

From an operational environment perspective, the device should function normally within a temperature range of 0–45 °C and possess resistance to various electromagnetic interferences. The Table 1 below summarizes these requirements.

#### 3.2.2. Software

The core component of the system’s software, the fatigue level classification model, must classify fatigue levels into five categories using video, thermal imaging, and audio signals collected over one minute. The target accuracy for this classification is set at 80%. Additionally, as user data accumulates, the system should enable personalized retraining to improve diagnostic accuracy. The summarized performance requirements are shown in Table 2 below.

The integrated platform must support APIs (Application Programming Interfaces) to facilitate communication with the current collector and to support the development of future upgraded applications. These APIs should include functions for user authentication, data transmission from the collector, fatigue measurement result requests, and receiving user feedback results.

**Figure 2:**
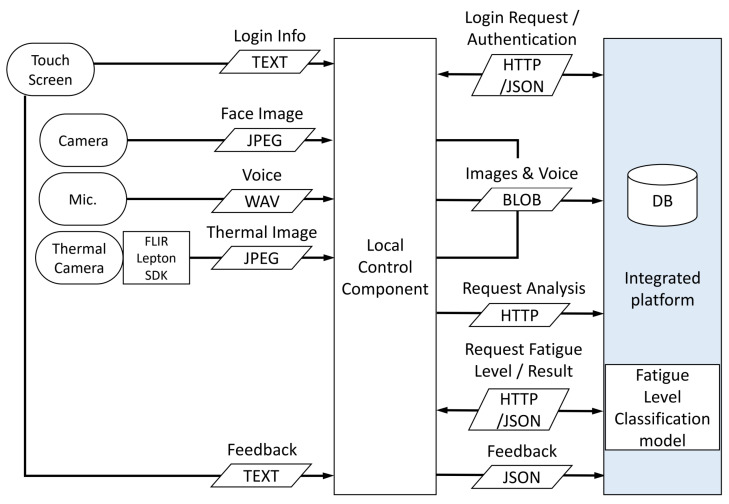
Data flow and API usage flowchart.

Figure 2 illustrates the data flow within the system. The data and request flows marked in blue on the right represent the communication with the integrated platform, where the endpoints correspond to the APIs of the integrated platform.

### 3.3. Fatigue Data for Classifier

The data used to train the fatigue level classification model was collected from professionals in occupations where physical fatigue during duty could result in loss of life or large-scale accidents [62]. These occupations include Air Force fighter pilots, police officers, firefighters, nurses, and truck drivers. All participants underwent training on the Bioethics and Safety Act for biometric data collection, provided their voluntary consent, and signed informed consent forms before data collection. The total dataset comprises approximately 14,550 sets, with each data set including audio (WAV), optical image (JPG), and thermal image (JPG) files. The dataset consists entirely of Asians aged 20 to 50.

Fatigue, while varying in degree, is a psychophysiological phenomenon experienced by all individuals and is not a clearly defined concept. Since “fatigue level” is a subjective judgment made by individuals, this study applied a “true fatigue value extraction” procedure during the initial data training phase to minimize subjective bias and improve the accuracy of fatigue level determination.

The process of extracting the true fatigue value involves the following steps [63,64]:

1 In the first step, participants indicate their current condition as a percentage and then assign a reverse fatigue grade. This allows for simple verification of gross errors.
2 In the second step, the reported grades are compared with the scores and grades derived from a separately developed survey-based multidimensional fatigue scale.
3 Finally, the reliability of the data are confirmed by comparing the grades with physiological markers, such as cortisol levels and other abnormal physiological responses, to finalize the fatigue levels.

Through the process of obtaining ground truth for fatigue, approximately 18% of the total 14,550 datasets we collected were excluded. The finalized fatigue levels were then used to train and validate algorithms utilizing video, thermal imaging, and audio data.

## 4. Implementation Results of the Fatigue Measurement System

### 4.1. Hardware Development Results

The main hardware components of the collector include a processor for data input and output, a video camera for biometric data collection, a thermal imaging camera, a microphone, a touch display for visualization and interaction, a battery for power supply, and a case to integrate all these components. The product names and key specifications used in this study are summarized in Table 3.

For the processor, the latest single-board computer, LattePanda Sigma, was chosen to ensure smooth operation. This processor not only delivers excellent performance but also comes with an integrated Wi-Fi module and Ethernet port, and its compact size (102 × 146 mm) makes it suitable for integration into the collector. The video camera was selected for its 5-megapixel resolution and compact size, allowing it to be placed near the thermal imaging camera. The microphone is a high-sensitivity condenser pin microphone with an integrated sound card capable of filtering ambient noise. The thermal imaging camera is the same model used during the collection of training thermal imaging data. The display is a 7-inch touch display specifically designed for LattePanda Sigma. For the battery, a model capable of 19V output and over two hours of operation was selected to power the LattePanda Sigma. The case was custom-designed and manufactured using a 3D printer, allowing all components to be integrated into a single unit.

**Figure 3:**
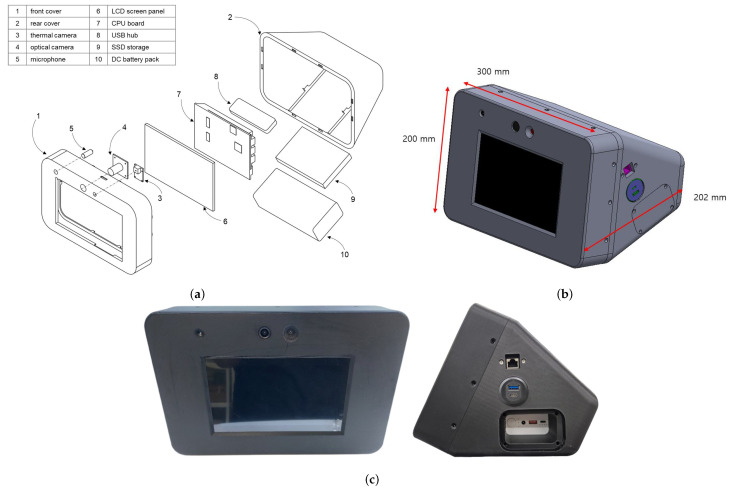
Design and implementation of the Data Collector. (**a**) Assembly diagram of key components. (**b**) 3D design. (**c**) Front and side views of final product.

The overall shape of the collector features the display screen positioned centrally, with the microphone, video camera, and thermal imaging camera located adjacent to one another above it. On the right side, there are an Ethernet port, external USB ports, and a battery cover. The dimensions are 300 mm in width, 200 mm in height, and 202 mm in depth, making it suitable for placement on a desk. Figure 3 presents the design and implementation of the collector. Figure 3a illustrates the assembly of the main components, Figure 3b showcases the 3D design, and Figure 3c provides the front and side views of the final product.

The case consists of two major parts: the front and back panels. The holes for the externally exposed microphone and cameras were designed to minimize gaps. Comparing Figure 3b,c confirms that the final product was manufactured precisely according to the design.

### 4.2. Software Development Results

**Figure 4:**
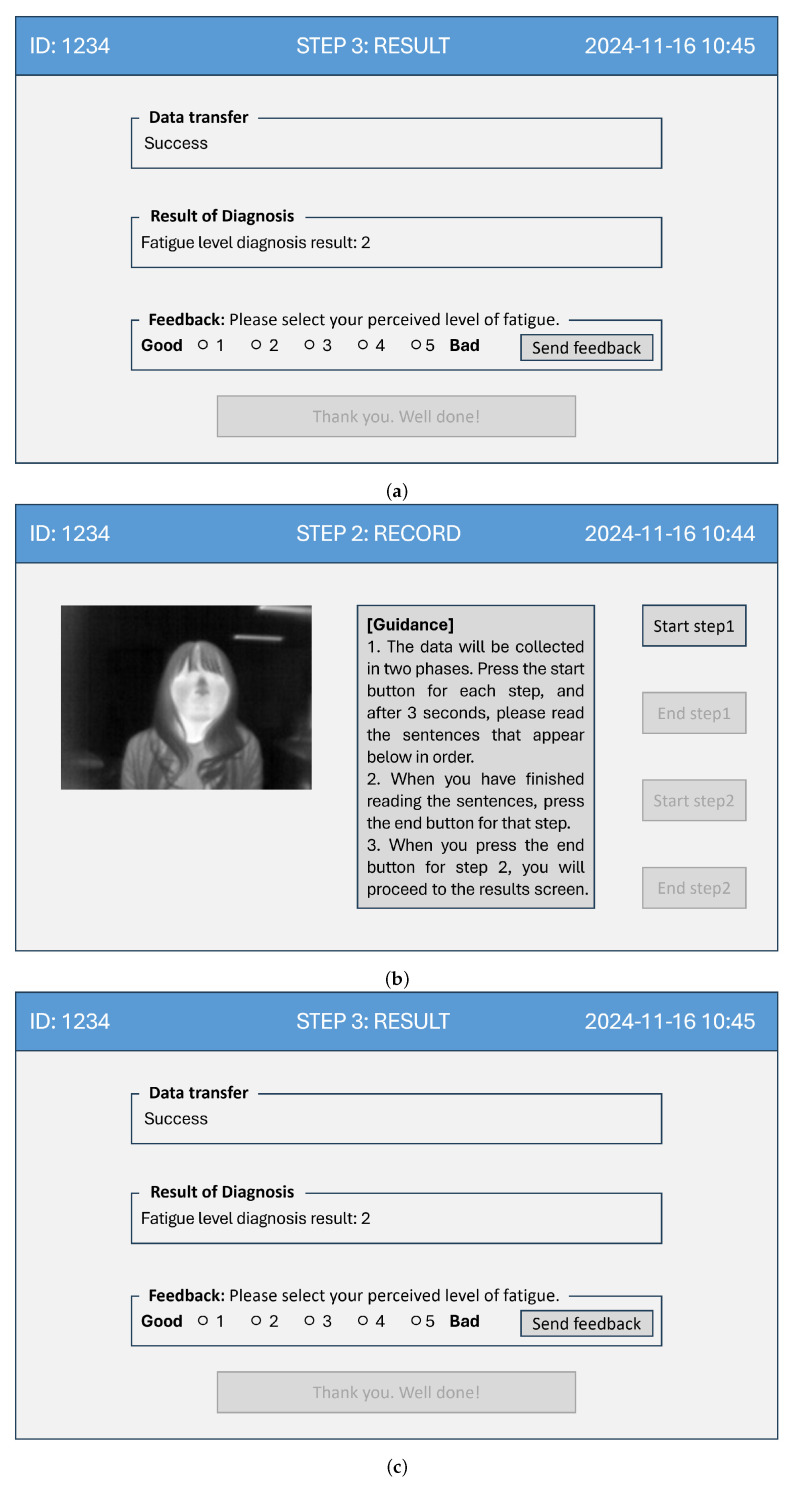
User interface examples of the data collector. (**a**) Login screen. (**b**) Data collection screen. (**c**) Results display screen.

The biometric data collector serves as the user-side terminal and can be operated via a touch-based screen or by connecting a keyboard and mouse through the USB port. The initial screen presented to the user is the login screen, as shown in Figure 4a. Since the system allows for personalized retraining of the fatigue level classification model based on the user’s data, login is used to identify the user. Upon successful login, the system proceeds to Figure 4b, which displays a thermal imaging screen to verify that the collector’s sensors are functioning properly.

Thermal imaging is used instead of color video captured by the optical camera to reduce the user’s psychological burden of seeing their face during data collection, thereby enabling more accurate fatigue diagnosis. Once data collection is complete, the third screen displays the fatigue level diagnosis results. As shown in Figure 4c, this screen presents the data transmission status and the fatigue diagnosis results. It also allows the user to send their feedback to the integrated platform.

### 4.3. Fatigue Level Classification Model

Once the collection of the three types of data are completed by the collector, the data are transmitted to the integrated platform, where the fatigue level classification model begins the analysis. The model utilizes SubNet to process and reduce the input data while preserving its meaning, addressing the significant differences in shape and size across various input modalities such as image, voice, and thermal data. By employing SubNets for modality embedding, the issue of input data inequality is effectively managed.

**Figure 5:**
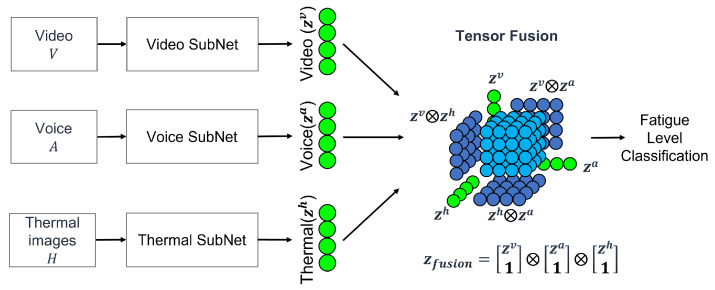
Fatigue level classifier model architecture.

The integrated model employs a method based on multi-modality tensor fusion, as illustrated in Figure 5 [65]. This approach combines the features extracted by each SubNet from the input data to classify the fatigue level.

For video data processing using SubNet, the input video is decomposed into frames. Each frame’s channels are expanded using DenseNet, followed by a Global Average Pooling (GAP) layer to reduce the frames into a one-dimensional vector. For voice data, the SubNet applies a simple Fully Connected Layer (FCL) to process the input data, compressing it while preserving its original information. Similarly, for thermal image data, the SubNet employs the same approach as video data processing, generating a processed input tensor for the thermal image data [65].

Our experiments are intended to classify fatigue levels 1-to-5, and all models were learned using cross-entropy loss. Adam was applied as an optimizer, and learning was conducted for 100 Epoch with a batch size of 16.

To train the classifier model, a total of 4690 datasets were used. This was determined by analyzing the final fatigue levels obtained in Section 3.3, where the fatigue ground truth with the smallest number of datasets had 938 samples. To ensure data balance, 938 samples were randomly selected for each fatigue level, from level 1 to level 5. As a result, 938 samples per fatigue level were used, leading to a total of 938 × 5 = 4690.

## 5. System Test Results

### 5.1. Experimental Results of Fatigue Level Classification

The core functionality of the system lies in accurately diagnosing fatigue levels, and the validation process is as follows. The input data consists of a separate dataset of 100 sets that were not used during training. From this dataset, 20 sets are randomly selected for testing. As previously described, each data set comprises audio, optical video, and thermal imaging data recorded over approximately one minute. The output values are classified fatigue levels, ranging from 1 to 5. This process is repeated five times, and the average accuracy is calculated.

Accuracy is determined by comparing the predicted fatigue levels with the true fatigue levels. If the predicted value matches the true value, it is classified as “true”; otherwise, it is classified as “false”. The accuracy is expressed as the percentage of “true” results out of the total test instances. This is defined by Equation (1) as follows:

(1) $$\mathrm{Accuracy}=\frac{\mathrm{Number}\;\mathrm{of}\;\mathrm{true}}{\mathrm{Total}\;\mathrm{Number}\;\mathrm{of}\;\mathrm{tests}}$$

As shown in Table 4, the average accuracy achieved is 89%. This is 9 percentage points higher than the target performance of 80%, demonstrating exceptional results. The testing of the fatigue level classification model is conducted at a certified testing institution.

### 5.2. Experimental Results on Personalized Learning

An experiment is conducted to verify the effectiveness of personalized learning in improving the accuracy of individual fatigue level measurements. Specifically, the study examines whether retraining the model based on user feedback on fatigue level results could enhance accuracy.

For the experiment, retraining is performed using 20 data sets per individual for a total of five participants. After retraining, another 20 data sets per individual, which are not used during training, are used for testing. The accuracy is calculated following the same method as defined in Equation (1).

As shown in Table 5, the average accuracy improved by 11 percentage points compared to the pre-retraining results. This experiment is also conducted at a certified testing institution.

### 5.3. Hardware Test Results

The hardware performance tests of the developed data collector were also conducted at a certified testing institution. The test agencies, criteria, and results for each item are summarized in Table 6, and all test criteria were satisfied.

First, the measurement distance test determines the operational range at which the fatigue level classification results can be demonstrated by the collector. The data collector, connected to the internet via wireless or wired communication, is fixed at a point more than 1 m away from the test subject. Fatigue level measurement is performed, and the displayed fatigue level is confirmed on the screen. The process is repeated 10 times in total, 5 times for each communication method. The measurement distance is validated based on whether the fatigue level is correctly displayed on the collector screen at a distance of 1 m for each communication method (wired/wireless). The communication method test follows the same procedure as the measurement distance test and verifies proper operation for both communication methods.

The power supply test measures the operating time through a battery discharge test. The collector is placed in a temperature- and humidity-controlled chamber, and the time until the device powers off is measured at 25 °C over 3 h. The results confirm that the collector operates for the full 3 h test period.

The operating temperature test includes two subtests. The first is the storage test, where the collector is powered off and placed in a temperature- and humidity-controlled chamber, maintained at 0 °C for 1.5 h and 45 °C for 1.5 h. Afterward, the device is inspected for exterior deformation, cracks, or operational issues. The second subtest is the operational test, where the collector is powered on and connected to a standard AC power supply while placed in the chamber. It is then operated for 1.5 h at 0 °C and another 1.5 h at 45 °C. The results confirm normal operation for both the storage and operational tests.

The electromagnetic interference (EMI) test includes conductive disturbance tests through external power supplies, radiative disturbance tests at specific frequencies, and various immunity tests. The specific types of tests are detailed in Table 7. All eight tests result in a determination of compliance.

## 6. Conclusions

This paper presents a novel contactless fatigue diagnosis system tailored for high-risk professions where physical and mental fatigue poses significant risks to safety and performance. By leveraging multimodal sensor data—video, thermal imaging, and audio—with an AI-driven classification model, the system offers a reliable and efficient solution for fatigue level monitoring. Experimental results reveal an average classification accuracy of 89%, surpassing the target benchmark. Additionally, the adaptive retraining mechanism enhances personalized diagnostic precision by incorporating user feedback, achieving an improvement of 11 percentage points in accuracy. The system’s hardware has been rigorously validated for reliability under diverse conditions, including temperature fluctuations and electromagnetic interference, ensuring robust performance in real-world applications. This innovative approach overcomes the limitations of traditional fatigue assessment methods by providing a non-invasive, real-time solution that is compatible with high-stress environments such as aviation, emergency response, and healthcare.

## Figures and Tables

**Table 1:** Detailed hardware requirements.

Item	Performance Requirements
Non-contact Biometric Data Acquisition	Capable of collecting video, audio, and thermal imaging data
Measurement Distance	At least 1 m (collector-to-subject)
Communication Method	Wi-Fi, Ethernet
Power Supply	Standard AC (220 V), operable for over 2 h with battery
Operating Temperature	0–45 °C
Electromagnetic Interference Test	Conducted/Radiated Emission Test, Electrostatic Discharge,
	Radiated RF Electromagnetic Field, Surge,
	Conducted RF Electromagnetic Field, Voltage Dip & Momentary Interruption

**Table 2:** Detailed software requirements.

Item	Performance Requirements
Fatigue Level Classification Grades	Distinguish between 1 and 5 levels
Biometric Signals for Classification	Non-contact biometric signals: video, audio, thermal imaging
	Input duration: Less than 1 min
Classification Accuracy	At least 80%
Individual Biometric Characteristics	Ability to reflect individual fatigue levels and biometric signal characteristics,
	improving accuracy when applied

**Table 3:** Hardware components of the collector.

Component	Model Name	Key Specifications
Processor	LattePanda Sigma	12 cores, 4.6 GHz, 32 GB memory
Video Camera	OV5640	5 MP, 2592 × 1944 resolution, 30 fps
Microphone	Britz BE-STM30U	Sensitivity: −30 dB, SNR: 74 dB
Thermal Camera	FLIR Lepton 3.5	160 × 120 resolution, 8.7 fps
Display	eDP for LattePanda Sigma	7-inch, 1024 × 600 resolution, touch-enabled
Battery	YTC 65 W	30,000 mAh, 19 V/4 A
Case	Custom-made	Desk-mounted, integrated design

**Table 4:** Accuracy results of fatigue level classification.

No.	Accuracy (%)
1	85
2	90
3	90
4	95
5	85
**Average**	**89.0**

**Table 5:** Accuracy results before and after personalized learning.

ID	Accuracy Before Retraining (%)	Accuracy After Retraining (%)
1	80	90
2	85	90
3	85	100
4	80	90
5	80	95
**Average**	**82.0**	**93.0 (+11 pp)**

**Table 6:** Performance requirement test results.

Test Item	Test Criteria	Test Results
Measurement Distance	At least 1 m	Criteria Met (1 m)
Communication Method	2 Types	Criteria Met (2 Types)
Collector Power	Battery discharge for over 2 h	Criteria Met (Over 3 h)
Operating Temperature	Storage and operational temperatures 0–45 °C	Criteria Met (Normal operation)
EMI Test	Normal operation before and after electromagnetic testing (8 types)	Criteria Met (Normal operation)

**Table 7:** EMI test results.

Test Item	Test Result
Main Power Port Conducted Disturbance Test	Pass
Radiated Disturbance Test Below 1GHz	Pass
Radiated Disturbance Test Above 1GHz	Pass
Electrostatic Discharge Immunity Test	Pass
Radiated RF Electromagnetic Field Immunity Test	Pass
Surge Immunity Test	Pass
Conducted RF Electromagnetic Field Immunity Test	Pass
Voltage Dips and Short Interruptions Immunity Test	Pass

## Data Availability

Data are not publicly available due to privacy.
